# Measuring Regional Eco-Efficiency in China (2003–2016): A “Full World” Perspective and Network Data Envelopment Analysis

**DOI:** 10.3390/ijerph17103456

**Published:** 2020-05-15

**Authors:** Weizhen Ren, Zilong Zhang, Yueju Wang, Bing Xue, Xingpeng Chen

**Affiliations:** 1 Key Laboratory of Western China’s Environmental Systems (Ministry of Education), College of Earth and Environmental Sciences, Lanzhou University, Lanzhou 730000, China; renwzh18@lzu.edu.cn (W.R.); yjwang2013@lzu.edu.cn (Y.W.); chenxp@lzu.edu.cn (X.C.); 2 Institute of Green Development for the Yellow River Drainage Basin, Lanzhou University, Lanzhou 730000, China; 3 Institute for Circular Economy in Western China, Lanzhou University, Lanzhou 730000, China; 4 Key Lab of Pollution Ecology and Environmental Engineering, Institute of Applied Ecology, Chinese Academy of Sciences, Shenyang 10016, China; xuebing@iae.ac.cn; 5 Institute for Advanced Sustainability Studies (IASS), 14467 Potsdam, Germany

**Keywords:** full-world model, eco-efficiency, network DEA model, GWR model

## Abstract

Eco-efficiency enhancement is an inherent requirement of green development and an important indicator of high-quality development in general. It aims to achieve the coordinated development of nature, the economy, and society. Therefore, eco-efficiency measurements should focus on not only total factor input, but also process analysis. Based on the “full world” model in ecological economic theory, this study constructed a theoretical framework for a composite economic-environmental-social system that reflects human welfare and sustainability. To this end, using network data envelopment analysis (DEA), this study established a staged eco-efficiency evaluation model that uses economic, environmental, and social factors to measure the overall and staged eco-efficiency of China’s provinces from 2003 to 2016 and analyze its spatiotemporal characteristics. A geographically weighted regression (GWR) model was also used to analyze the influencing factors of eco-efficiency changes and the spatial differentiation in their effect intensity. The findings were as follows: (1) China’s overall eco-efficiency is still at a low level. It varies significantly from region to region, and only three regions are at the frontier of production. The eastern region has the highest eco-efficiency, followed by the central region, and the gap between the central and western regions has gradually narrowed. In terms of staged efficiency, the level of eco-efficiency in the production stage is less than in the environmental governance stage, which is less than that in the social input stage. (2) In terms of the efficiency of each stage, the efficiency level of the production stage showed a downward trend throughout the entire process, and the decline in the central and western regions was more obvious. The social input stage and the environmental governance stage both showed upward trends. The social input stage showed a higher level, and the increase was relatively flat during the period of study. Efficiency continued to rise during the environmental governance stage from 2003 to 2010 and rose overall, but with some fluctuations from 2011 to 2016. (3) Geographically weighted regression showed that the effects of the influencing factors on eco-efficiency had obvious spatial heterogeneity. The factors affecting overall, production stage, and social input eco-efficiency were, in order of effect intensity from high to low, economic growth level, marketization level, and social input level. In terms of environmental governance, social input level had the greatest impact, followed by economic growth; marketization level did not show a significant impact.

## 1. Introduction

After experiencing a period of rapid economic growth, China has entered a new stage of development. In the context of the “new normal” of the economy and the advancement of ecological civilization, eco-efficiency research can provide ideas for solving the contradiction between economic development and resource depletion, while also promoting regional sustainable development. First proposed by Schaltegger and Sturm [1] and then promoted by the World Business Council for Sustainable Development (WBCSD), the eco-efficiency concept has been widely recognized by society [2]. In 1998, the Organization for Economic Cooperation and Development (OECD) extended the eco-efficiency concept to governments, industrial enterprises, and other organizations. Eco-efficiency also refers to the ability of ecological resources to meet human needs, and it is a typical input–output process [3]. In terms of concepts and index systems, recent studies have mainly explored the eco-efficiency of research objects from the perspective of eco-efficiency connotations [4,5]. Such index systems involve three-dimensional scales of economic, social, and ecological environments, with resource and environment factors mainly used as inputs and economic value or regional output used as expected outputs [6,7]. Meanwhile, in terms of eco-efficiency measurement, current methods include energy analysis [8,9], the index system method [10], material flow analysis [11,12], the ecological footprint method [13,14], and data envelopment analysis [15,16,17,18]. In addition, studies on different objects at different levels of eco-efficiency have covered products [19,20], enterprises [21,22], industries [23,24], and regions [25,26,27]. At the regional scale, the current research of eco-efficiency mainly focusses on spatiotemporal changes, convergence analysis, and its influencing factors in different countries [28,29,30].

By reviewing the literature, we found that the general view of eco-efficiency is that it seeks to obtain high economic output with low resource consumption, environmental cost, and ecological damage. However, the existing eco-efficiency connotations and measurement standards do not fully reflect the essential needs of human development (i.e., sustainable welfare levels). Firstly, in terms of the connotation of eco-efficiency, research has expanded from initial analyses of economic output under environmental impacts to the study of the relationship between social services and the growth rate of ecological loads [31]. Despite this, the existing research has not fully emphasized the importance of people in the entire ecosystem. Secondly, in terms of index systems and final outputs, research has expanded from a resource-and-environment orientation to include three-dimensional indicators of economy, society, and the environment [32,33]. While evaluation systems have gradually improved, human welfare factors have been neglected in measuring the final outputs of human activity. Researchers have mainly used economic indicators such as gross domestic product (GDP) as the final output [34], but that indicator is only a stage indicator in the operation of the ecological economic system. In terms of developing a composite economic–environmental–social system, the ultimate goal of sustainable development is to benefit humanity [35]; however, the imbalance or trade-off between rapid economic growth and environmental protection have led to considerable negative impacts on people’s quality of life in some areas of China [36]. It is necessary, therefore, to add indicators to measure social benefits or welfare.

It is clear that the existing research has not sufficiently extended the connotation of eco-efficiency, improved the evaluation index system, or reached a consensus on the final output of the eco-economic system. The present study, therefore, starting from the connotation of eco-efficiency, takes the Human Development Index (HDI) as the final output goal to construct a three-dimensional index system of economic production, environmental governance, and social input. Based on the network DEA model, this study measures the characteristics of the eco-efficiency of each province in mainland China from 2003 to 2016 and analyzes the causes of heterogeneity using a geographically weighted regression model.

## 2. Methods and Data

### 2.1. Index System Construction and Data Description

**Figure 1:**
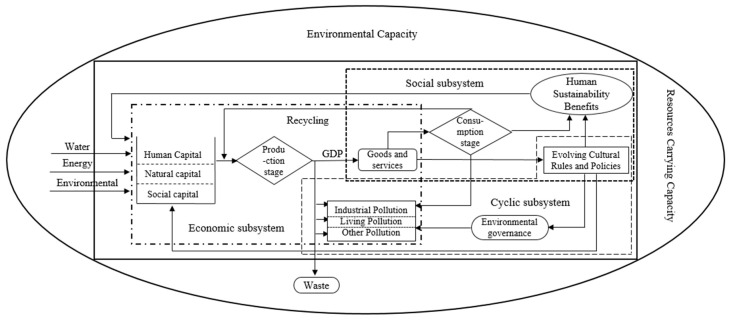
Economic–environmental–social composite system [40].

To reflect interprovincial eco-efficiency in China, this study constructed a composite economic–environmental–social framework (Figure 1) that reflects human welfare and sustainability, based on the relevant literature [37,38] as well as OECD ideas about eco-efficiency [39]. The framework also considers the United Nations’ “Changing our World: The 2030 Agenda for Sustainable Development” and draws upon Robert Costanza’s “full world” model for ecological economy [40]. In the proposed composite system, nature provides the environmental capacity for human development. The economic subsystem inputs natural, human, and social capital and other production factors to produce the target GDP, while controlling the corresponding pollution and ecological damage. Figure 1 shows that the process from factor input to welfare formation can be divided into three stages. The first is the production stage, corresponding to the economic subsystem. The inputs at this stage include natural, human, and social capital; the outputs are goods and services. The measurement indicator is GDP. The second is the environmental governance stage, corresponding to the circulation subsystem. This stage aims to reduce the negative effects of pollutant emissions on environmental capacity and resource carrying capacity during the production and consumption stages. The third is the social input stage, corresponding to the social subsystem. Here, the cultural rules, systems, and policies of the composite system will reinvest the human and social capital needed in the production stage to improve production efficiency as well as environmental governance. The human social system achieves maximum, sustainable welfare effectiveness through consumption activities (of products, services, and the natural environment) and through the evolution of rules, systems, and policy guarantees.

Based on the theoretical framework and the available data, this study chose the end-of-year number of employees, capital stock, total energy consumption, and total public water consumption as the initial input indicators. The intermediate output indicators included both the expected intermediate output GDP and the undesired intermediate output (wastewater, waste, and solid waste). In the environmental treatment stage, investment in environmental pollution treatment was used as an input factor to measure the input used to improve the ecological environment for each area. The urban sewage treatment rate, air quality of major cities, and utilization rate of solid waste were used as output indicators. For the social input stage, the proportions of R&D input and social public expenditure were selected as input variables. HDI was used as the final output indicator of the composite system. Table 1 shows the index composition.

The data sources are shown in Table 1. Thirty provinces, autonomous regions, and municipalities (Tibet was excluded due to missing data) were included. The GDP of each province in the past year was treated as a constant price in 2000. Capital stock was calculated according to the perpetual inventory method [41]. Due to changes in statistical caliber, the number of employees in Guizhou Province has undergone parallel iterations since 2006. For 2016, the industrial waste gas emissions of individual provinces were fitted using the trend-line method. When calculating HDI, since there were censuses for only 2000 and 2010, as well as 1% population sample survey data every five years, the life expectancy indicator for the remaining years was replaced with the average life expectancy during similar years. In terms of education indicators, due to limited data availability, the comprehensive gross enrollment rate was replaced by student attendance, which can reflect the level of education in China to some extent.

### 2.2. Research Methods

#### 2.2.1. Network DEA Model

The DEA model, which can better measure and evaluate the relative effectiveness of decision-making units (DMUs) with multiple inputs and outputs, has been widely applied in measuring eco-efficiency [42]. However, the traditional DEA method treats the production system as a “black box”, ignoring the internal structure of the system and its interacting relationships. Furthermore, it has failed to achieve the efficiency evaluation of complex production systems with multistage correlation. The slacks-based network DEA model (network SBM), which was proposed by Tone and Tsutsui, fully considers the relationships between subprocesses and can simultaneously give values for system efficiency and subprocess efficiency and also has a greater advantage in terms of evaluating the efficiency of complex production systems associated with multiple stages [43]. Therefore, by dividing the ecosystem into three stages (i.e., economic production, environmental governance, and social input), this study constructed a network SBM model that considers undesired outputs. The efficiency of DMU_o_ (o refers to the number of DMU, O=1,…,n) can be calculated by solving the following linear program [44]:

(1) $$\begin{array}{ccc} \frac{1}{\rho_{0}^{*}} & = & \max{\sum_{k=1}^{k}W}^{k}\left[1+\frac{1}{r_{{}^{k}}}\left(\sum_{r=1}^{r_{k}}\frac{s_{ro}^{k+}}{y_{ro}^{k}}\right)\right]\\ s.t.x_{o}^{k} & = & X^{k}\lambda^{k}+s_{o}^{k-}\\ y_{o}^{k} & = & Y^{k}\lambda^{k}-s_{o}^{k+}\\ e\lambda^{k} & = & 1\\ \lambda^{k} & \geq & 0,s_{o}^{k-}\geq 0,s_{o}^{k+}\geq 0,(\forall k) \end{array}$$

where $\rho_{0}^{*}$ represents the overall efficiency of DMU_o_, $w^{k}$ is the relative weight of division *k*, and $s^{k-}(s^{k+})$ are the input (output) slack vectors.

#### 2.2.2. Geographically Weighted Regression (GWR) Model

Based on the spatial non-stationarity [45], the geographically weighted regression (GWR) model uses geographical coordinates and core functions to perform a local regression estimation on each group of spatially adjacent subsamples. It is an effective analysis method for studying spatial heterogeneity. The model is as follows [46]:

(2) $$y_{i}=\beta_{o}(\mu_{i},v_{i})+\sum_{k=1}^{p}\beta_{k}(\mu_{i},v_{i})x_{ik}+\varepsilon_{i}$$

where $y_{i}$ represents the eco-efficiency of ith province, $\left(\mu_{i},v_{i}\right)$ is the spatial geographic coordinates of ith province, $\beta_{k}$$\left(\mu_{i},v_{i}\right)$ is the regression coefficient of the kth explanatory factor in province *i*, and $\varepsilon_{i}$ is the error term.

## 3. Results

DEA-SOLVER-PRO14d was used to solve the model, calculate efficiency, and obtain the overall eco-efficiency of 30 provinces in China from 2003 to 2016, as well as the input–output efficiency of three major regions (east, middle, and west), during the production, social input, and environmental governance stages.

### 3.1. Overall Eco-Efficiency

The national average eco-efficiency of 30 provinces in China from 2003 to 2016 was 0.8478, indicating that China’s eco-efficiency still needs improvement. It also varied significantly from region to region (Table 2). During the study period, only three provinces and cities were on the frontier of production, accounting for 10% of the total sample; 14 regions exceeded the average level, accounting for 46.67% of the total sample. In terms of the average level, the eastern region had the highest eco-efficiency, followed by the central region and the western region. The average eco-efficiency of the eastern region was 0.1346 higher than that of the central region and 0.1543 higher than that of the western region. Tianjin, Shanghai, and Hainan, located in the eastern region, had an effective status, followed by Beijing, Guangdong, Jiangsu, Shandong, Fujian, Zhejiang, Hebei, and Liaoning. Anhui, in the central region, had the highest eco-efficiency, followed by Heilongjiang, Henan, Hunan, Hubei, Jilin, Shanxi, and Jiangxi. For the western region, Qinghai was in the leading position, followed by Ningxia, Chongqing, Yunnan, Guangxi, Sichuan, Inner Mongolia, Gansu, Xinjiang, Guizhou, and Shaanxi. In terms of regional spatial differences, the differences in the levels of eco-efficiency in the western region were greater than those of the eastern region, whose eco-efficiency was greater than that of the central region.

**Figure 2:**
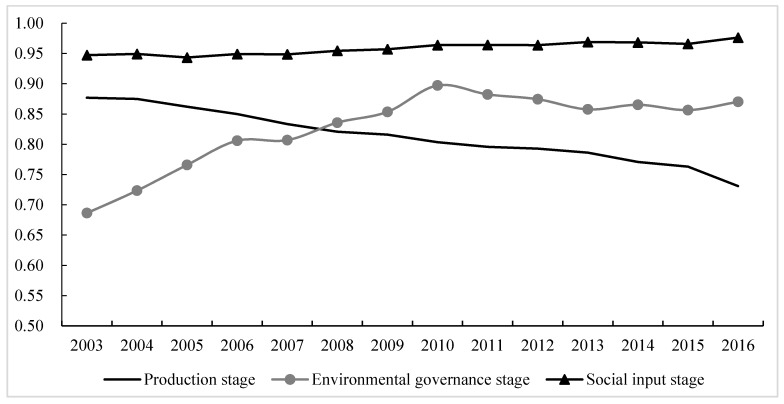
Evolution of eco-efficiency in the three major stages in China from 2003 to 2016.

Comparing the average efficiency of the three stages (Figure 2), eco-efficiency in the social input stage from 2003 to 2016 showed a slowly rising trend and was always higher than in the production and environmental governance stages. During the study period, the average efficiency of the production stage decreased year on year and started to become lower than that in the environmental governance stage in 2008. The environmental governance stage showed a rapid upward trend from 2003 to 2010 and slowly developed, with fluctuations, from 2011 to 2016.

### 3.2. Staged Eco-Efficiency

**Figure 3:**
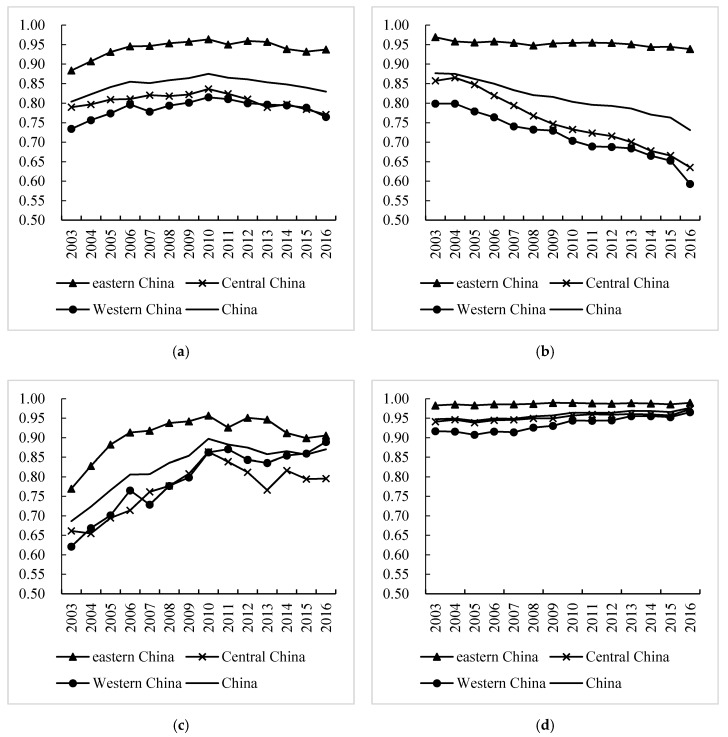
(**a**) Overall efficiency; (**b**) production stage efficiency; (**c**) efficiency of environmental governance stage; (**d**) efficiency of social input stage.

Figure 3a–d shows the evolution of China’s overall eco-efficiency and the input–output efficiency in three stages from 2003 to 2016. Regarding overall efficiency, the eastern region, with the highest eco-efficiency, far surpassed the western and central regions and showed a rising trend. There was little difference between the central and western regions, and the western region continuously approached the central region (Figure 3a).

For the production stage (Figure 3b), overall, China’s economic efficiency was still at a low level, with an average of 0.8126. During the study period, only five provinces and cities nationwide were at the frontier of production, and all were in the eastern region. No province or city in the central or western region had achieved DEA effectiveness. Thirteen regions exceeded the average level, including 10 in the eastern, one in the central, and two in the western regions. For the change in time scale, whether the whole country or the three regions, eco-efficiency in the production stage showed a downward trend. Among the regions, the gap between the central and western regions and the eastern region is relatively obvious, and this gap is constantly expanding. The eastern region had the highest level of eco-efficiency, with an average efficiency value above 0.95. The efficiency values in the central and western regions were significantly lower, with values of 0.7535 and 0.7155, respectively.

At the stage of environmental governance (Figure 3c), overall, China’s eco-efficiency was at a low level, with an average of 0.8273. Of the 14 provinces and municipalities that exceeded the national average, nine, one, and four were in the east, central, and western regions, respectively. Tianjin, Shanghai, and Hainan in the east were at the frontier of production. Beijing achieved DEA effectiveness during 2005–2016, Fujian during 2009–2016, and Guangdong during 2010–2016. Only Anhui in the central region reached DEA effectiveness (2015–2016). Qinghai in the western region was at the frontier of production; Chongqing achieved DEA effectiveness during 2007–2016 and Guizhou and Ningxia during 2010–2016. In terms of time changes, the eco-efficiency values of China’s three major regions during the environmental governance stage increased in varying degrees, with the eastern region maintaining a leading position. The average efficiency of the western region started to surpass that of the central region in 2011 and tended to be on par with the average efficiency across the country. At this stage, the improvement in the eco-efficiency value reflects the fact that China has paid more and more attention to environmental protection in recent years, and has made remarkable achievements in environmental governance [47].

At the social input stage, the average efficiency of the 30 provinces in China from 2003 to 2016 was 0.9586, which was higher than that of the production stage and environmental governance stage as a whole. During the study period, the provinces with effective efficiency during the social input stage included three regions—Tianjin, Shanghai, and Hainan—which only accounted for 10% of the total sample, indicating that most provinces and cities had redundant resources or insufficient output. From a time series perspective, the average values for the eco-efficiency of the three major regions all showed upward trends in varying degrees, and the specific performance was as follows: eastern region > central region > western region. However, the gaps were not significant (see Figure 3d).

In general, China’s eco-efficiency was relatively stable overall from 2003 to 2016, and there was no obvious upward trend. The eco-efficiency values in the three stages showed regular changes. During the entire process, the efficiency level in the production stage showed a downward trend, and the decline in the central and western regions was more pronounced. Both the social input stage and the environmental governance stage showed upward trends. Eco-efficiency in the social input stage increased relatively slowly during the study period. The efficiency of the environmental governance stage was on the rise during 2003–2010 and continued to rise, with fluctuations, from 2011 to 2016. At the regional level, there were large differences between and within regions, and there were fewer provinces and cities at the frontier of production. Currently, China’s eco-efficiency is still at a low level.

The analysis showed that, for the eastern region, which has a high degree of marketization and a high economic level, whether in terms of overall eco-efficiency or the efficiency of the three stages, the level was higher than in the central and western regions. Especially in the production stage, which is closely related to marketization degree and economic growth level, the gap is even more obvious. For the environmental governance and social input stages, which mainly depend on government input and intervention, the gap was significantly reduced. Therefore, it is necessary to analyze the effects of marketization degree, economic growth, and social input on overall eco-efficiency and the efficiency of the three stages.

## 4. Influencing Factors

Open GeoDa was used to analyze the spatial auto-correlation of China’s provinces in 2016. It can be seen that the Moran’s I was 0.2031 and the coefficient was significant at the 5% level. Therefore, it is necessary to conduct further heterogeneity analyses. To explore the abovementioned effects, a GWR model was used to analyze the cross-sectional data of China’s provincial eco-efficiency in 2016. With reference to the previous literature, this study selected per capita GDP (hereafter, Pgdp) to measure the level of economic growth, the proportion of non-state-owned fixed-asset investments among the total regional fixed-asset investments to measure the degree of marketization (hereafter, Market), and the proportion of social fiscal expenditure to GDP to measure social input level (hereafter, Investment). To reasonably calculate the regression model, the standard deviation of each index was first processed, then the collinearity test was performed, and all variables passed the test. The results obtained using the GWR model were analyzed as shown in Table 3. According to the median and quartile values, the regression fitting estimates of different quantiles are significantly different, indicating that the effects of each explanatory variable on the differences in provincial eco-efficiency are heterogeneous. The average value reflects the average level of the contribution of influencing factors to the provincial level of eco-efficiency. From the average value of the contribution of each influencing factor to the overall level of eco-efficiency in China, as well as the eco-efficiency in the production and the social input stages, the regression coefficients are sorted in descending order as Pgdp > Market > Investment. For the environmental governance stage, the order is Investment > Pgdp > Market; thus, the market has no significant impact on the environmental governance stage.

**Figure 4:**
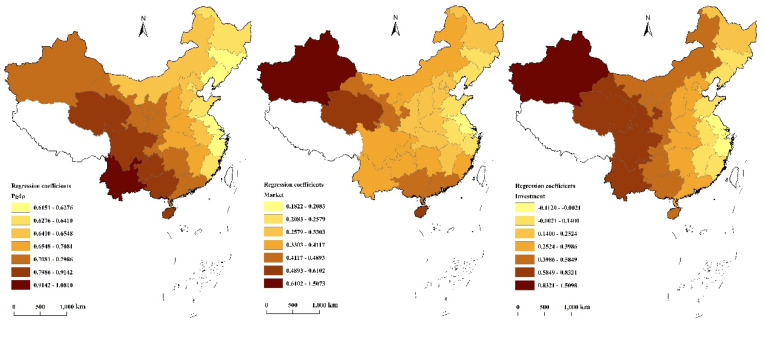
Spatial distribution of regression coefficients of the GWR model of overall eco-efficiency in China’s provinces.

The spatial distribution of the regression coefficients of the three independent variables of the GWR model was plotted using ArcGIS (Pgdp, Market, and Investment from left to right). Figure 4 shows the results. 

It can be seen in Figure 4 that economic growth (Pgdp) had the most significant and positive effect on the overall level of eco-efficiency in China’s provinces, and the overall spatial pattern gradually increases from the northeast to southwest. The high-value areas of the regression coefficients were mainly distributed in southwestern regions such as Yunnan, Qinghai, and Sichuan. These regions have low levels of economic development, and their eco-efficiency is at low and medium levels, indicating that an increase in per capita GDP will have a positive effect on the level of eco-efficiency. Therefore, an appropriate increase in per capita GDP will help improve the development level of eco-efficiency. The low-value areas were mainly concentrated in the northeast and the Yangtze River Delta. Their per capita GDP is high, the level of eco-efficiency is at high and medium levels, and the role of economic growth in promoting eco-efficiency is relatively weak.

The marketization degree (Market) positively affected the eco-efficiency of a province as a whole, indicating that a Market increase plays a positive role in promoting eco-efficiency. The promotion effect is seen in the overall spatial pattern of the Yangtze River Delta, which is a low-value center, and the value increases geographically toward the surrounding areas. Areas with high values for the regression coefficients are mainly concentrated in Xinjiang, Qinghai, Gansu, Hainan, and Guangdong. Among them, the degree of marketization is relatively low in Xinjiang, Qinghai, and Gansu. Their local governments should therefore take measures to increase marketization to improve eco-efficiency. In places such as Hainan and Guangdong, although the marketization degree is already high, the level of eco-efficiency still has room for improvement. Continuing to expand the degree of marketization will play a significant role in promoting eco-efficiency. The low-value areas are mainly located in the Yangtze River Delta, and their marketization degree is relatively high. Continuing to increase the degree of marketization will not have a significant impact on the level of eco-efficiency in that region.

The effect of social input level (Investment) on overall eco-efficiency shows complex spatial heterogeneity, with the effect going both ways—that is, both promoting and inhibiting (the regression coefficient is positive or negative). Overall, the effect increases from east to west. Areas with high values for the regression coefficients are mainly concentrated in western regions such as Xinjiang, Qinghai, Gansu, and Yunnan. Eco-efficiency in this region is at low and medium levels, the level of economic development is low, and social-fiscal investment accounts for a high proportion of the GDP; thus, continued increases in fiscal investment will have a positive effect. The low-value areas are mainly concentrated in the eastern coastal areas, among which Jiangsu, Zhejiang, and Shanghai are negative areas, indicating that the region has a high degree of economic development. Continuing to increase the level of financial investment will not have a particularly positive effect, or even a negative effect.

**Figure 5:**
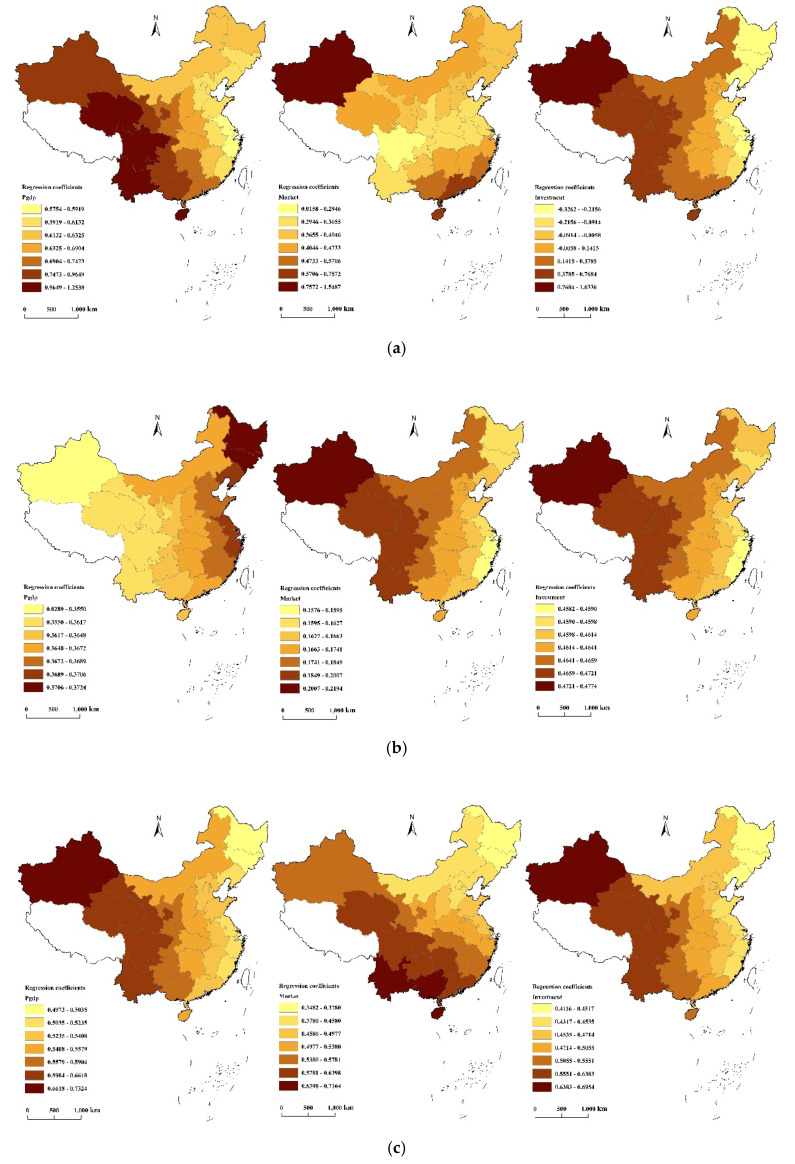
GWR regression analysis of China’s provincial-level eco-efficiency by stage; (**a**) GWR regression result for efficiency at the production stage; (**b**) GWR regression result for efficiency at the environmental governance stage; (**c**) GWR regression result for efficiency at the social input stage.

Figure 5 shows the results of the GWR model regression analysis of staged efficiency. In terms of efficiency during the production stage (Figure 5a), both economic growth and marketization degree appear to have a promotion effect. The promotion effect of economic growth factors in the western and central regions is stronger, while it is weaker in the eastern region. The effect intensity of marketization shows a spatial pattern wherein it is high in the southeast coastal area and northwest Xinjiang, but low in the middle. In a similar manner to overall eco-efficiency, the effect of social input level on production stage efficiency shows large spatial heterogeneity. In some provinces and autonomous regions along the east coast and in the northeast, an increase in social input level will inhibit the improvement of production stage efficiency, while in the west it will promote improvement.

For the efficiency of the environmental governance stage (Figure 5b), the three influencing factors all show promotion effects. The promotion effect of economic growth is the strongest in the eastern region, and it decreases gradually in the central and then western regions. The promotion effect intensity of the social input level is the highest in the western region, followed by the central region, and it is the lowest in the eastern region. The marketization factor did not pass the significance test, which also shows that market measures for environmental governance have not played a significant role at the present stage.

As for the efficiency of the social input stage (Figure 5c), the three influencing factors all show promotion effects. The promotion effect intensity of economic growth and social input levels is high in the west, followed by the middle, and it is the lowest in the east. The intensity of marketization shows a spatial pattern of gradual decline from southwest to northeast.

## 5. Discussion

In this study, we tried to build a composite economic-environmental-social framework from the full-world perspective for eco-efficiency evaluation, and used HDI, selecting human wellbeing as the final output indicator of the system, which is different from previous studies that used GDP as the final output. In terms of overall eco-efficiency, China is still at a low level, and it varies significantly from region to region. With the development of China’s economy, the pressure on the ecological environment continues to rise. It is important, therefore, for China to pursue sustainable economic development with low inputs, low emissions, and high outputs. The comparison of the efficiency values of the substage and the composite system showed that, during the study period, the eco-efficiency level of the production stage at the provincial level in China was less than that of the environmental governance stage, whose eco-efficiency level was less than that of the social input stage; the production and environmental governance stages were the leading causes of low regional eco-efficiency and regional differences. The production stage is particularly critical, indicating that the resource and environmental pressures generated during the economic production stage in the system’s operation are relatively large. At present, China’s environmental load mainly comes from the production stage.

In terms of spatial distribution, there were obvious spatial differences in the eco-efficiency of the 30 provinces and cities. Only Shanghai, Tianjin, and Hainan were at the frontier of production. Other provinces and cities had relatively inefficient states to varying degrees. Overall, the eastern region far surpassed the western and central regions, and the central and western regions showed little difference. The western region approached or even surpassed the development trend of the central region. Regarding regions, differences in the levels of eco-efficiency in the western region were greater than in the eastern region, whose eco-efficiency was greater than that of the central region. Therefore, when improving eco-efficiency in the central and western regions, attention should be paid to balancing the development of provinces in the region, and effort should be made to reduce eco-efficiency gaps between provinces.

In terms of the efficiency of each stage, the overall efficiency level in the production stage showed a downward trend. The gap between the efficiency levels of the central and western regions and the eastern region was obvious—and that gap is getting wider. The eco-efficiency level of the eastern region was the highest, and that of the central and western regions was lower than the national average and had decreased significantly. Important measures for improving regional eco-efficiency include adjusting and optimizing the economic development mode of the central and western regions and increasing the economic output per unit of ecological environment load. Overall eco-efficiency in the environmental governance stage was rising, with the eastern region maintaining a leading position. With advancements in western development and increased investment in ecologically fragile areas, the average efficiency of the western region has gradually surpassed that of the central region and is on par with that of the whole country. Due to the relatively developed industry in the region, it is densely populated, and the pressure on resources and the environment is large; thus, the imbalance between economic development and the ecological environment is more prominent. In the social input stage, the average eco-efficiency of the 30 provinces during the study period was higher than that of the production stage and the environmental governance stage. The average eco-efficiency values of the three major regions all showed upward trends to varying degrees, which manifested as eastern region > central region > western region; however, the difference was not significant. This reflects the importance of people in the composite economic–resource–social system, emphasizing the welfare of people in the social stage.

Regarding the driving factors of eco-efficiency, except for marketization, which had no significant effect on the environmental governance stage, the other explanatory variables showed significant spatial heterogeneity in their effects on overall eco-efficiency and staged efficiency. Economic growth and marketization showed a positive promotion effect on overall eco-efficiency and staged efficiency, and this effect had regular spatial differentiation characteristics. The level of social input had a more complex effect on overall eco-efficiency and efficiency at the production stage. In the eastern coastal areas and parts of the northeast, it had a negative inhibiting effect while in the central and western regions, it showed a positive promotion effect. In terms of the efficiency of the environmental governance stage and the social input stage, the level of social input showed a positive promotion effect. The influence of social input presented a clear spatial pattern, being low in the east and high in the west, which is exactly the opposite of China’s current economic pattern. It is necessary, then, to grasp the relationship between economic development, marketization degree, public input level, resources and the environment in order to improve China’s eco-efficiency.

This study attempted to use the network DEA method and the "full-world" framework to break the previous "black box" model of eco-efficiency evaluation, but due to the availability of data and data quality, the study had certain limitations. In this article, we analyzed an overall macro system, and some aspects, especially the corresponding indicators of the industry, have not been broken down in detail. For China, the types of industries and regions are more diverse. Input indicators with different geographic characteristics and different types of resources will produce different outputs. Therefore, it is critical to pay attention to this aspect. This is also the focus of our next study.

## 6. Conclusions

Based on the “full-world” model proposed by Robert Costanza, this study constructed a new model framework for evaluating eco-efficiency by applying the network SBM model, and explored the influence factors of eco-efficiency at a provincial level, i.e., economic development, social input and marketization, using GWR models. The results indicate that China’s overall ecological efficiency is still at a low level, and the overall distribution pattern is high in the east and low in the west; the efficiency level in the production stage is declining, while the stage of environmental governance and social input are showing an increasing trend. The effects of different influencing factors on changes in eco-efficiency show obvious spatial heterogeneity. The regions with high levels of economic development and marketization tend to have higher level of eco-efficiency.

## Figures and Tables

**Table 1:** China’s interprovincial ecological efficiency evaluation index system.

Stage and Node		Variable and Unit	Data Source
Economic production stage	Input variable	Number of employees at the end of the year (10,000 people)	*China Statistical Yearbook*
		Capital stock (100 million yuan)	*China Statistical Yearbook*
		Total energy consumption (10,000 tons of standard coal)	*China Energy Statistical Yearbook*
		Total water used by the whole society (100 million cubic meters)	*China Environmental Statistics Yearbook*
		GDP (100 million yuan)	*China Statistical Yearbook*
	Output variable	Wastewater discharge (10,000 tons)	*China Statistical Yearbook*
		Industrial waste gas emissions (100 million cubic meters)	*China Environmental Statistics Yearbook*
		Solid waste emissions (10,000 tons)	*China Statistical Yearbook*
Environmental governance stage	Input variable	Investment amount for environmental pollution treatment (100 million yuan)	*China Environmental Statistics Yearbook*
		Municipal sewage treatment rate	*China Environmental Statistics Yearbook*
	Output variable	Air quality in major cities	*China Environmental Statistics Yearbook*
		Comprehensive utilization rate of solid waste	*China Statistical Yearbook*
Social input stage	Input variable	Proportion of R & D technology investment	*China Statistical Yearbook of Science and Technology*
		Social expenditure (100 million yuan)	*China Statistical Yearbook*
	Output variable	Human Development Index	*China Statistical Yearbook*

**Table 2:** Overall eco-efficiency values by province from 2003 to 2016.

DMU	2003	2007	2010	2013	2016	Average	Ranking
Beijing	0.9224	0.9938	0.9969	0.9977	0.9991	0.9847	4
Tianjin	1.0000	1.0000	1.0000	1.0000	1.0000	1.0000	1
Hebei	0.7022	0.8380	0.8747	0.8913	0.7724	0.8080	17
Liaoning	0.7272	0.7878	0.8304	0.8159	0.7351	0.7769	21
Shanghai	1.0000	1.0000	1.0000	1.0000	1.0000	1.0000	1
Jiangsu	0.9246	0.9412	0.9740	0.9424	0.9725	0.9562	7
Zhejiang	0.8861	0.9570	0.9717	0.9375	0.9667	0.9489	10
Fujian	0.9104	0.9477	0.9664	0.9590	0.9555	0.9520	9
Shandong	0.8571	0.9914	0.9898	0.9899	0.9159	0.9552	8
Guangdong	0.7895	0.9558	0.9974	0.9941	0.9952	0.9618	6
Hainan	1.0000	1.0000	1.0000	1.0000	1.0000	1.0000	1
Eastern Region	0.8836	0.9466	0.9637	0.9571	0.9375	0.9403	(1)
Jilin	0.7937	0.7485	0.7736	0.8138	0.8519	0.7753	22
Shanxi	0.7508	0.8214	0.8168	0.7397	0.6502	0.7736	23
Jiangxi	0.5771	0.7155	0.8257	0.7703	0.6994	0.7402	24
Anhui	0.9355	0.8916	0.8872	0.8433	0.8414	0.8760	12
Henan	0.8077	0.8518	0.8431	0.7660	0.7994	0.8170	15
Hubei	0.7799	0.8100	0.8269	0.7737	0.7393	0.7949	20
Hunan	0.7778	0.8303	0.8569	0.7693	0.8007	0.8132	16
Heilongjiang	0.8953	0.8959	0.8604	0.8402	0.7839	0.8554	14
Central Region	0.7897	0.8206	0.8363	0.7895	0.7708	0.8057	(2)
Chongqing	0.7932	0.8780	0.9039	0.9139	0.8601	0.8602	13
Sichuan	0.6332	0.7579	0.7970	0.6843	0.6727	0.7384	25
Guizhou	0.3144	0.6541	0.8159	0.8266	0.6707	0.6951	29
Yunnan	0.9324	0.7359	0.8125	0.7745	0.7195	0.8042	18
Shaanxi	0.5500	0.6995	0.7333	0.7179	0.7152	0.6780	30
Gansu	0.6287	0.6921	0.7308	0.8177	0.7744	0.7239	27
Qinghai	1.0000	0.9630	0.9821	1.0000	1.0000	0.9810	5
Ningxia	0.9277	0.9067	0.9117	0.8782	0.8679	0.9146	11
Xinjiang	0.7747	0.6921	0.6924	0.6793	0.5782	0.7153	28
Inner Mongolia	0.6942	0.7575	0.7749	0.7250	0.7521	0.7380	26
Guangxi	0.8265	0.8246	0.8109	0.7442	0.7988	0.7969	19
Western Region	0.7341	0.7783	0.8150	0.7965	0.7645	0.7860	(3)
National average	0.8037	0.8513	0.8752	0.8535	0.8296	0.8478	

**Table 3:** Descriptive statistical analysis of the local regression coefficients of the geographically weighted regression (GWR) model.

Stage	Variable	Average	Standard Deviation	Min	Max	Upper Quartile	Median	Lower Quartile	Significance Level
Overall	Pgdp	0.725	0.1147	0.6151	1.0810	0.7880	0.6751	0.6365	***
	Investment	0.3643	0.3231	−0.1120	1.5098	0.5118	0.3025	0.1222	*
	Market	0.3788	0.2344	0.1822	1.5073	0.3965	0.3272	0.2743	*
Production stage	Pgdp	0.7364	0.1733	0.5754	1.2538	0.8518	0.6531	0.6071	***
	Investment	0.1924	0.4095	−0.3262	1.6336	0.3989	0.1208	−0.1198	*
	Market	0.4568	0.2289	0.0158	1.5487	0.4575	0.3884	0.3599	**
Environment governance stage	Pgdp	0.3661	0.0039	0.0289	0.3724	0.3688	0.3667	0.3640	*
	Investment	0.4639	0.0043	0.4582	0.4774	0.4658	0.4632	0.4607	*
	Market	0.1740	0.0136	0.1576	0.2194	0.1791	0.1714	0.1641	/
Social input stage	Pgdp	0.5614	0.0507	0.4973	0.7324	0.5826	0.5515	0.5294	***
	Investment	0.5024	0.0692	0.4116	0.6954	0.5399	0.4872	0.4528	*
	Market	0.5378	0.0898	0.3482	0.7164	0.5980	0.5347	0.4693	**

Note: *, **, and *** indicate significance at the levels of 0.1, 0.01, and 0.001, respectively/indicates significance at the level of 0.25.
